# Supplementary material for: Modelling Skylarks (Alauda arvensis) to Predict Impacts of Changes in Land Management and Policy: Development and Testing of an Agent-Based Model
Source: PLoS One. 2013 Jun 6;8(6):e65803. doi: 10.1371/journal.pone.0065803 (PMC3675089; doi:10.1371/journal.pone.0065803)
Supplement: Supporting Information S4 — The skylark ODdox as a zipped archive. (ZIP) [file pone.0065803.s004.zip › Skylark_ODdox/class_skylark___female-members.html]

ALMaSS Skylark ODdox: Member List


ALMaSS Skylark ODdox
 2.0


- Main Page
- Related Pages
- Classes
- Files

- Class List
- Class Index
- Class Hierarchy
- Class Members

Skylark\_Female Member List

This is the complete list of members for Skylark\_Female, including all inherited members.

|  |  |  |
| --- | --- | --- |
| AddStriglingMort(int lifestage) | Skylark\_Base | inline |
| Age | Skylark\_Base |  |
| BeginStep(void) | Skylark\_Female | virtual |
| BSuccess | Skylark\_Adult | protected |
| CalcFoodTime(double target) | Skylark\_Female | protected |
| CalculateEggNumber() | Skylark\_Female | protected |
| CheckForFields() | Skylark\_Female | protected |
| CheckManagement(void) | TAnimal |  |
| CheckManagementXY(int x, int y) | TAnimal |  |
| CopyMyself(int a\_sktype) | Skylark\_Adult | virtual |
| Skylark\_Base::CopyMyself() | TAnimal | inlinevirtual |
| CorrectWrapRound(void) | TAnimal | inlineprotected |
| CurrentStateNo | TALMaSSObject |  |
| DailyMortality(int mort) | Skylark\_Base | protected |
| Dying() | TAnimal | inlinevirtual |
| EggCounter | Skylark\_Female | protected |
| EndStep(void) | Skylark\_Female | virtual |
| EstablishTerritory() | Skylark\_Female |  |
| FeedYoung() | Skylark\_Female | protected |
| GetBadWeather() | Skylark\_Adult | protected |
| GetFood(int time) | Skylark\_Female | protected |
| GetMigrationMortality() | Skylark\_Female | protected |
| GetVegHindrance(int PolyRef) | Skylark\_Adult | protected |
| GetWeatherHindrance() | Skylark\_Adult | protected |
| GoodWeather | Skylark\_Adult | protected |
| InSquare(rectangle R) | Skylark\_Base |  |
| KillThis() | TAnimal | inlinevirtual |
| m\_aTerrlist | Skylark\_Adult | protected |
| m\_Born\_x | Skylark\_Base |  |
| m\_Born\_y | Skylark\_Base |  |
| m\_BreedingAttempts | Skylark\_Female | protected |
| m\_BreedingSuccess | Skylark\_Female | protected |
| m\_CIPEGrid | Skylark\_Female |  |
| m\_Counter1 | Skylark\_Female | protected |
| m\_CurrentSkState | Skylark\_Base |  |
| m\_EggNumber | Skylark\_Female | protected |
| m\_Location\_x | TAnimal | protected |
| m\_Location\_y | TAnimal | protected |
| m\_MinFemaleAcceptScore | Skylark\_Female | protected |
| m\_MyHome | Skylark\_Base |  |
| m\_NestTime | Skylark\_Female | protected |
| m\_OurLandscape | TAnimal | protected |
| m\_OurPopulationManager | Skylark\_Base |  |
| m\_OurTerritories | Skylark\_Base |  |
| m\_pcide\_conc | Skylark\_Base |  |
| m\_pesticide\_accumulation | Skylark\_Base |  |
| m\_pesticide\_sprayed | Skylark\_Female | protected |
| m\_pesticide\_sprayed\_die | Skylark\_Female | protected |
| m\_Size | Skylark\_Base |  |
| m\_toowet | Skylark\_Female | protected |
| MyClutch | Skylark\_Female | protected |
| MyExtractEff | Skylark\_Adult | protected |
| MyMate | Skylark\_Female |  |
| MyTerritory | Skylark\_Adult | protected |
| NestLoc | Skylark\_Female | protected |
| On\_FoodSupply(double) | Skylark\_Base | inlinevirtual |
| OnArrayBoundsError() | TALMaSSObject |  |
| OnBreedingSuccess() | Skylark\_Female |  |
| OnBreedSuccess() | Skylark\_Female | inline |
| OnBroodDeath() | Skylark\_Female |  |
| OnClutchDeath() | Skylark\_Female |  |
| OnEggsHatch() | Skylark\_Female |  |
| OnFarmEvent(FarmToDo event) | Skylark\_Female | protectedvirtual |
| OnMaleNeverComesBack(Skylark\_Male \*AMale) | Skylark\_Female |  |
| OnMateDying() | Skylark\_Female |  |
| OnMateHomeless() | Skylark\_Female |  |
| OnSetMyClutch(Skylark\_Clutch \*p\_C) | Skylark\_Female | inline |
| OnStopFeedingChicks() | Skylark\_Female |  |
| Paired | Skylark\_Adult |  |
| PesticideResponse() | Skylark\_Female | protectedvirtual |
| RemoveEM(double food) | Skylark\_Adult | protectedvirtual |
| ResetBreedingSuccess() | Skylark\_Female |  |
| Resources | Skylark\_Female | protected |
| SensibleCopy() | Skylark\_Female |  |
| SetX(int a\_x) | TAnimal | inline |
| SetY(int a\_y) | TAnimal | inline |
| Skylark\_Adult(int x, int y, double size, int age, SkTerritories \*Terrs, Landscape \*L, Skylark\_Population\_Manager \*SPM, int bx, int by, int mh) | Skylark\_Adult |  |
| Skylark\_Base(int x, int y, SkTerritories \*Terrs, Landscape \*L, Skylark\_Population\_Manager \*SPM, int bx, int by, int mh) | Skylark\_Base |  |
| Skylark\_Female(int x, int y, double size, int age, SkTerritories \*Terrs, Landscape \*L, Skylark\_Population\_Manager \*SPM, int bx, int by, int mh) | Skylark\_Female |  |
| Spiral(int x, int y, int radius) | Skylark\_Female | protected |
| Spiral2(int x, int y, int radius) | Skylark\_Female | protected |
| st\_Arriving() | Skylark\_Female | protected |
| st\_BuildingUpResources() | Skylark\_Female | protected |
| st\_CaringForYoung() | Skylark\_Female | protected |
| st\_Dying() | Skylark\_Female | protected |
| st\_EggHatching() | Skylark\_Female | protected |
| st\_Emigrating() | Skylark\_Female | protected |
| st\_Finding\_Territory() | Skylark\_Female | protected |
| st\_Floating() | Skylark\_Female | protected |
| st\_Flocking() | Skylark\_Female | protected |
| st\_GivingUpTerritory() | Skylark\_Female | protected |
| st\_Immigrating() | Skylark\_Female | protected |
| st\_Incubating() | Skylark\_Female | protected |
| st\_Laying() | Skylark\_Female | protected |
| st\_MakingNest() | Skylark\_Female | protected |
| st\_PreparingForBreeding() | Skylark\_Female | protected |
| st\_StartingNewBrood() | Skylark\_Female | protected |
| st\_StoppingBreeding() | Skylark\_Female | protected |
| st\_TempLeavingArea() | Skylark\_Female | protected |
| Step(void) | Skylark\_Female | virtual |
| StepDone | TALMaSSObject |  |
| Supply\_BreedingAttempts() | Skylark\_Female |  |
| Supply\_BreedingSuccess() | Skylark\_Female |  |
| Supply\_m\_Location\_x() | TAnimal | inline |
| Supply\_m\_Location\_y() | TAnimal | inline |
| Supply\_NestTime() | Skylark\_Female | inline |
| SupplyFarmOwnerRef() | TAnimal |  |
| SupplyMyClutch() | Skylark\_Female | inline |
| SupplyPolygonRef() | TAnimal | inline |
| SupplyPosition() | TAnimal |  |
| TALMaSSObject() | TALMaSSObject |  |
| TAnimal(int x, int y, Landscape \*L) | TAnimal |  |
| WhatState() | Skylark\_Base | inlinevirtual |
| ~Skylark\_Adult() | Skylark\_Adult |  |
| ~TALMaSSObject() | TALMaSSObject | virtual |


- Generated on Thu Jan 10 2013 13:15:37 for ALMaSS Skylark ODdox by
   1.8.1.1
